# Effects of Head and Neck Position on Nasotracheal Tube Intracuff Pressure: A Prospective Observational Study

**DOI:** 10.3390/jcm10173910

**Published:** 2021-08-30

**Authors:** Hye Jin Kim, Jaewon Jang, So Yeon Kim, Wyun Kon Park, Hyun Joo Kim

**Affiliations:** Department of Anesthesiology and Pain Medicine, Anesthesia and Pain Research Institute, Yonsei University College of Medicine, 50-1 Yonsei-ro, Seodaemun-gu, Seoul 03722, Korea; jackiedi@yuhs.ac (H.J.K.); JAEWONJANG2020@yuhs.ac (J.J.); KIMSY326@yuhs.ac (S.Y.K.); WKP7ARK@yuhs.ac (W.K.P.)

**Keywords:** intratracheal/instrumentation, intubation, intratracheal/adverse effects, pressure

## Abstract

To prevent endotracheal tube-related barotrauma or leakage, the intracuff pressure should be adjusted to 20–30 cm H_2_O. However, changes in the nasotracheal tube intracuff pressure relative to neck posture are unclear. In this study, we investigated the effect of head and neck positioning on nasotracheal tube intracuff pressure. Fifty adult patients with nasotracheal tubes who were scheduled for surgery under general anesthesia were enrolled. Following intubation, intracuff pressure was measured by connecting the pilot balloon to a device that continuously monitors the intracuff pressure. Subsequently, the intracuff pressure was set to 24.48 cm H_2_O (=18 mmHg) for the neutral position. We recorded the intracuff pressures based on the patients’ position during head flexion, extension, and rotation. The initial intracuff pressure was 42.2 cm H_2_O [29.6–73.1] in the neutral position. After pressure adjustment in the neutral position, the intracuff pressure was significantly different from the neutral to flexed (*p* < 0.001), extended (*p* = 0.003), or rotated (*p* < 0.001) positions. Although the median change in intracuff pressure was <3 cm H_2_O when each patient’s position was changed, overinflation to >30 cm H_2_O occurred in 12% of patients. Therefore, it is necessary to adjust the intracuff pressure after tracheal intubation and each positional change.

## 1. Introduction

After tracheal intubation for general anesthesia, the intracuff pressure of the tracheal tube should be adjusted to 20–30 cm H_2_O [1]. Insufficient cuff inflation may lead to air leakage, inability to achieve adequate tidal volume in mechanical ventilation, and ventilator-associated pneumonia due to aspiration of contaminated oropharyngeal secretions [2]. Conversely, an excessively inflated cuff causes ischemia by pressing against the capillaries in the mucous membrane of the inner wall of the trachea, increasing the risk of complications such as sore throat, tracheal stenosis, tracheal rupture, and nerve injury [3,4]. Therefore, the cuff must be appropriately inflated so that it contacts the tracheal wall at a reasonable pressure.

The intracuff pressure does not remain constant during surgery and varies due to several factors such as the patient’s body temperature, airway pressure, endotracheal intubation time, and anesthesia with nitrous oxide [4,5,6,7,8]. Moreover, the head-down position and pneumoperitoneum caused by carbon dioxide insufflation can lead to increased airway and intracuff pressures [9,10]. A manual cuff pressure manometer can be used to set the initial intracuff pressure and adjusted relative to subsequent changes in the intracuff pressure. However, this method risks a pressure drop at each measurement, potentially resulting in cuff underinflation [11]. Therefore, continuous cuff pressure regulation is preferable to intermittently measuring intracuff pressure using a manual manometer [12].

In head and neck surgery, the patient’s head and neck are positioned to facilitate the operation by exposing the surgical field. Moreover, intraoperative head and neck position changes are required in cases of surgery of several areas. The head and neck position can alter orotracheal tube intracuff pressure [13,14,15] since the airway’s length and dimension changes with neck flexion, extension, and rotation. This can cause tube displacement and cuff compression or release [16,17,18].

Similar to the orotracheal tube, a nasotracheal tube can move relative to head and neck positioning [19], leading to too-high or too-low intracuff pressures. However, the pressure changes within differently shaped nasotracheal tubes, and with the use of “soft” tubes to reduce nosebleeds, may differ from those observed with orotracheal tubes. Changes in the nasotracheal tube intracuff pressure relative to neck posture have not been researched. The Portex^®^ (Smiths Medical International, Hythe, UK) nasotracheal tube—considered the most malleable and widely used—is used in our hospital [20]. We observed changes in the nasotracheal tube intracuff pressure relative to the head and neck position using continuous cuff pressure monitoring. The primary study outcome was the intracuff pressure recorded during various head and neck postures.

## 2. Materials and Methods

### 2.1. Study Population

This prospective, single-center, observational study was approved by the Institutional Review Board of Severance Hospital, Yonsei University Health System (Seoul, Korea; numbers: 4-2020-0442; 9 June 2020). The study protocol was registered at www.clinicaltrials.gov (NCT04441970, 22 June 2020). Written informed consent was obtained from all patients participating in this study. This manuscript adheres to the Strengthening the Reporting of Observational Studies in Epidemiology guidelines (Checklist S1). From June 2020 to September 2020, adult patients aged ≥20 years scheduled to undergo surgery under general anesthesia and with a nasotracheal tube were enrolled. We excluded patients with cervical spine disease or a history of cervical spine surgery.

### 2.2. Anesthetic Management and Measurements

In the operating room, the patients placed their heads on a pillow in a supine position. Before inducing anesthesia, we evaluated head flexion, extension, and rotation. Standard monitors for pulse oximetry, three-lead electrocardiography, and non-invasive blood pressure measurement were attached. Anesthesia was induced using 1–2 mg/kg propofol, 0.5–1.0 µg/kg remifentanil, and 0.6–1.0 mg/kg rocuronium. Mask ventilation was performed using oxygen at 5 L/min and sevoflurane 2–4 vol%. After establishing a complete neuromuscular block, intubation was performed using a video laryngoscope with nasotracheal tubes with an internal diameter (ID) of 6.0–7.0 (Ivory PVC Portex^®^ North Facing Nasal Soft-Seal Cuffed Polar Preformed Endotracheal Tube, Smiths Medical International, Hythe, UK). Endotracheal intubation was confirmed by the presence of an end-tidal CO_2_ waveform and adequate breath sounds at lung auscultation. The tube was fixed on the nares using tape. The tube’s cuff was inflated by injecting air into the pilot balloon using a 10 mL syringe. The anesthesiologists assessed the adequacy of cuff inflation by palpating the pilot balloon with their fingers. The breathing circuit was connected to the tube, and mechanical ventilation was started in a volume-controlled mode. The target tidal volume was 8 mL/kg ideal body weight with a fresh gas flow of 2 L/min, respiratory rate of 12 breaths/min, an inspiratory-expiratory ratio of 1:2, and positive end-expiratory pressure of 5 cm H_2_O. After 30 s, intracuff pressure was measured by connecting the pilot balloon to a calibrated manometer for continuous intracuff pressure monitoring (TruWave^TM^ PX260, Edwards Lifesciences LLC, CA, USA). This monitoring device was connected to a separate workstation via a cable. When connected to the pilot balloon, the intracuff pressure value registered immediately [21]. All raw pressure data were initially measured in mmHg, then converted to cm H_2_O by multiplying by 1.36. Following this, the intracuff pressure was set at 24.48 cm H_2_O (=18 mmHg) in the neutral position. We measured the intracuff pressures during head flexion, extension, and rotation. Patients’ characteristics such as age, sex, height, weight, body mass index, American Society of Anesthesiologists physical class, and nasotracheal tube size were recorded.

### 2.3. Study Endpoints

The primary outcome of this study was the intracuff pressure at various head and neck postures.

### 2.4. Statistical Analysis

Using a singed-rank test, we compared the intracuff pressure observed while the head and neck were in the neutral position to the intracuff pressures when the head and neck were extended, flexed, or rotated. The magnitude of change in the intracuff pressure when the head was changed from a neutral position to a flexed, extended, or rotated position was also compared using a signed-rank test. The same statistical method was applied for changes in peak inspiratory pressure with head positioning. We compared the number of cases where the intracuff pressure decreased or increased and the number of cases where the intracuff pressure was outside the appropriate range of 20–30 cm H_2_O using McNemar’s test. Spearman’s correlations were performed to analyze the relationship between changes in peak inspiratory pressure changes and intracuff pressure changes with head positioning. Values are presented as mean ± SD, median [IQR], or numbers (percentages). Analyses were conducted using SAS software version 9.4 (SAS Institute Inc., Cary, NC, USA). A *p*-value of 0.016 was considered statistically significant. To adjust for multiple comparisons, we applied a Bonferroni correction.

### 2.5. Sample Number Calculation

The primary outcome was the nasotracheal tube intracuff pressure. To determine the sample size, we estimated the intracuff pressure based on a previous report [14]. A sample size of 38 achieved 80% power to detect a mean of paired differences of 0.5 with an estimated standard deviation of differences of 0.9 and with an alpha of 0.016 using a two-sided paired *t*-test. A total of 47 participants were required after accounting for a 20% dropout rate and 50 participants were enrolled in this study.

## 3. Results

**Figure 1:**
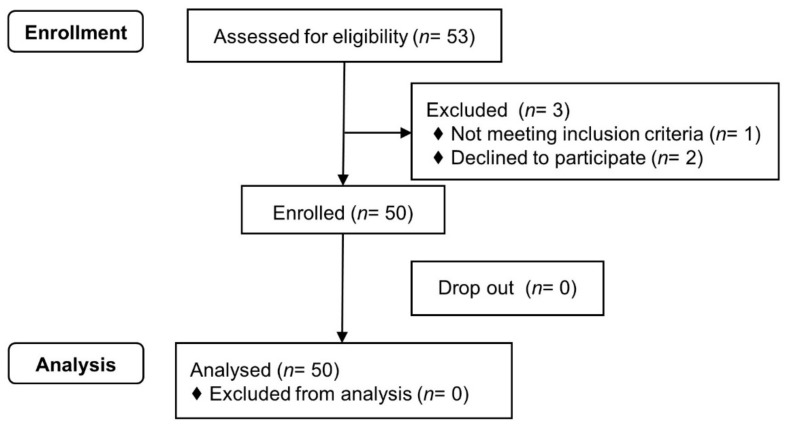
Flowchart of study patient enrollment.

Of the 53 patients screened, 50 patients were enrolled and completed the study (Figure 1), no missing data were recorded for any patient.

The patients’ baseline characteristics summarized in Table 1 and Table 2 show the initial intracuff pressure set by palpating the pilot balloon. The initial median intracuff pressure was 42.2 [29.6–73.1] cm H_2_O in the neutral position. Only 12% of patients had intracuff pressures between 20 and 30 cm H_2_O in the neutral position.

Table 3 documents the changes in intracuff pressure when the pressure was set as 24.48 cm H_2_O. The intracuff pressure was significantly different between neutral and flexed (*p* < 0.001), extended (*p* = 0.003), and rotated (*p* < 0.001) positions. These changes from the neutral position pressures were all <10 cm H_2_O. The number of patients with increases in intracuff pressure in the flexed, extended, and rotated positions was 40 (80%), 24 (48%), and 31 (62%), respectively. The number of patients with decreases in the intracuff pressure in the flexed, extended, and rotated positions was 3 (6%), 10 (20%), and 4 (8%), respectively. The number of patients with intracuff pressures >30 cm H_2_O in the flexed, extended, and rotated positions was 6 (12%), 2 (4%), and 3 (6%), respectively. No patient demonstrated intracuff pressures <20 cm H_2_O in any of the positions. Table 4 shows the changes in peak inspiratory pressure with head positioning. The maximum change in peak inspiratory pressure according to the head positioning was 1 cm H_2_O. There were no statistically significant correlations between changes in peak inspiratory pressure and changes in intracuff pressure with head positioning (Table 5).

## 4. Discussion

Head and neck positioning affects the nasotracheal tube intracuff pressure. When pilot balloon palpation was used to determine the intracuff pressure, the initial pressure was out-of-range in 88% of patients. When the initial intracuff pressure was adjusted to an appropriate value using a manometer, increases in intracuff pressure occurred most during neck flexion. Although the median change in the intracuff pressure was <3 cm H_2_O when the patient’s position was changed, 12% of patients experienced overinflation >30 cm H_2_O.

In this study, the nasotracheal tube intracuff pressure changed significantly from the neutral to flexed, extended, and rotated neck positions. Our novel finding agrees with that of a prior study, intracuff orotracheal tube pressures are affected by head and neck positioning [14,17]. Komosawa et al. found that, in adults, head flexion increased the intracuff pressure by a median of 10.3 cm H_2_O, head extension increased the pressure by a median of 5.0 cm H_2_O [14]. Additionally, in pediatric patients, Kako et al. noted that neck flexion increased intracuff pressure with the greatest magnitude of mean 6.9 cm H_2_O [17]. Similarly, in our study, intracuff pressure was most increased by neck flexion compared to the neck extension and rotation. In fact, in 80% of our patients, intracuff pressure increased with neck flexion.

The mechanism of this change in intracuff pressure by head and neck positioning has been suggested as tube migration and deformation of the shapes of adjacent anatomic structures [15,22]. The airway length changes as the head or neck posture changes and the tube is pulled outward or inward along the trachea [19]. Kim et al. observed orotracheal tube tip withdrawal from the carina with head rotation [13]. Jordi Ritz et al. showed that neck flexion moved the tube toward the carina and neck extension moved the tube away from the carina [18]. In addition, Kako et al. explained the cuff pressure changes as follows: Neck flexion displaces the trachea posteriorly, causing cuff compression by the esophagus and cervical spine, while neck extension moves the trachea anteriorly, causing cuff pressure release [17]. Therefore, we suggest that nasotracheal tube movements along the airway and the change in the force exerted on the cuff by surrounding structures are the reasons for intracuff pressure changes in different head and neck positions.

Nevertheless, in our study, the median change in intracuff pressure was minimal—<3 cm H_2_O–and most patients (88%) still demonstrated intracuff pressures of 20–30 cm H_2_O after changing positions. This finding contrasts with that of Komosawa et al., they found that neck flexion and extension caused intracuff pressure to exceed 30 cm H_2_O with high incidences of 90% (flexion) and 50% (extension) [14]. One potential reason for our observations of stable intracuff pressure for the nasotracheal tube could be that nasotracheal tubes move less during posture changes than orotracheal tubes. Tailleur et al. found that the orotracheal tube was displaced unpredictably with head position changes (even causing right main stem bronchial intubation at one point) [16]. Yamanaka et al. investigated the airway length changes during nasotracheal intubation in pediatric patients. They found that the head flexion-to-extension positional changes caused a slight change in the length of the pharyngeal cavity, the authors asserted that the cuff position was also likely to be changed [23]. Hartrey et al. found that the magnitude of movement was smaller with nasotracheal, compared to that with orotracheal tubes [19]. This may explain why the intracuff pressures in our study were relatively well-maintained. Moreover, Kim et al. noted that the degree of orotracheal tube displacement differed depending on where the tube was fixed [13]. Nasotracheal tubes are fixed in the nares, which are relatively closer to the center of the face, which will be on the head positioning axis. Therefore, even if the posture is changed, the pathway of the nasotracheal tube is likely to be well-maintained with minimal distortion. Moreover, the Portex^®^ nasotracheal tube is the most malleable and can bend easily [24], additionally, the cuff is highly elastic [24]. Matsuki et al. found that the ID of flexible Portex^®^ nasotracheal tube could be decreased from the cuff pressure at 20 cm H_2_O in 38 °C hot water. In contrast, no significant changes were observed with harder nasotracheal tubes such as the Mallinckrodt^TM^ Nasal RAE^TM^ tracheal tube and the Parker Flex-Tip^®^ Tracheal tube [25]. These characteristics may be helpful for compensating for intracuff pressure increases by compliant cuff expansion into an unobstructed direction [24].

Despite the small change in intracuff pressure, it is worth noting that the intracuff pressure exceeded 30 cm H_2_O in 12% of the cases according to the positional change. Seegobin and van Hasselt showed that intracuff pressure above 30 cm H_2_O impairs mucosal capillary blood flow [1]. In addition, Calder et al. showed that in pediatric elective day-case surgery, the incidence of sore throat significantly increased from 20% when the cuff pressure was 21–30 cm H_2_O to 68% when the cuff pressure was 31–40 cm H_2_O [26].

We additionally estimated the initial intracuff pressure with patients placed in a neutral head position when the cuff pressure was set by pilot balloon palpation (and without using a manometer). The pilot balloon palpation method is still the most commonly and simple method of determining cuff pressure [27]. The pressure exceeded the appropriate range in 88% of patients, and excess pressure was applied more often (74%) than less pressure. This finding is consistent with that of Giusti et al.’s study which showed that manual palpation accurately estimated cuff pressure in only 10% of participants [28]. Michlig et al. noted that only 34% of participants correctly determined that a cuff pressure of 120 cm H_2_O was too high, while 32% of participants misidentified the pressure as too low [29]. We thought that the pilot balloon palpation method would be more reliable with the Portex^®^ nasotracheal tube since the pilot balloon of the Portex^®^ nasotracheal tube has a relatively large diameter, this would improve tactile sensation according to Laplace’s law [30,31]. However, our results showed that—for the Portex^®^ nasotracheal tube—pilot balloon palpation did not accurately determine the cuff pressure. Excess cuff pressure can lead to serious complications such as tracheal injury or rupture [3]. Therefore, we do not recommend solely relying on the pilot balloon palpation method.

Our use of a manometer for continuous pressure monitoring is based on a previous study [21]. Continuous monitoring is recommended since pressure decreases when the manometer is attached or removed from the pilot balloon. Even if the pressure is within the appropriate range at the time of measurement, it may leave this range after measurement [11]. Aeppli et al. demonstrated that the cuff pressure drops occurred by disconnecting the manometer from the pilot balloon in about 80% of cases by 0.6 cm H_2_O, this phenomenon leads to low intracuff pressure (<20 cm H_2_O in 31% of cases) and increases the risk of tracheal aspiration [11]. Our use of a continuous monitoring device produced more accurate data than intermittent measurements could have.

This study had several limitations. First, we did not compare different tube types. The Portex^®^ nasotracheal tube has a malleable shaft and cylindrical polyvinyl chloride cuff. Other tubes with more-rigid tube shafts or conical cuffs or cuffs made with polyurethane material were also used [32]. Therefore, our results may not be applicable for different tube types. Second, the position changes of the tube were not observed. Third, our study enrolled adult patients. The smaller airways in children may produce larger changes in intracuff pressure, resulting in higher incidences of overinflation and underinflation [17].

## 5. Conclusions

Pilot balloon palpation is an unreliable means of controlling the nasotracheal tube intracuff pressure. If the initial intracuff pressure is accurately adjusted to within a safe range using a nanometer, in most cases minimal changes will be observed upon head and neck repositioning. However, there is no guarantee that the intracuff pressure will fall within the safe range in all patients when their postures changes. Thus, it is necessary to adjust the intracuff pressure after tracheal intubation and repositioning.

## Figures and Tables

**Table 1:** Patients’ characteristics.

Variable	Total (*n* = 50)
Age, y	35.2 ± 13.8
Sex (M/F)	28 (56%)/22 (44%)
Weight, kg	69.7 ± 15.1
Height, cm	168.4 ± 8.4
Body mass index, kg/m^2^	24.5 ± 4.3
Nasotracheal tube size (6/6.5/7), internal diameter (mm)	22/21/7
American Society of Anesthesiologists class, 1/2/3	39 (78%)/9 (18%)/2 (4%)

Data are presented as mean ± SD or numbers (percentages).

**Table 2:** Initial intracuff pressure in the neutral head position when the cuff pressure was set by pilot balloon palpation.

Parameter	Initial Neutral Position
Intracuff pressure (cm H_2_O)	42.2 [29.6–73.1]
Range of intracuff pressure (cm H_2_O)	15.0–178.2
Number of cuffs with intracuff pressure <20 cm H_2_O	7 (14%)
Number of cuffs with intracuff pressures 20–30 cm H_2_O	6 (12%)
Number of cuffs with intracuff pressures >30 cm H_2_O	37 (74%)

Data are presented as median [IQR] or numbers (percentages).

**Table 3:** Changes in the intracuff pressure with head positioning when the initial value was set as 24.48 cm H_2_O (=18 mmHg) using a pressure monitoring device.

Parameter	Flexion	Extension	Rotation
Intracuff pressure (cm H_2_O)	27.2 [25.8–28.6] ^a^	24.5 [24.5–27.2] ^a^	25.8 [24.5–27.2] ^a^
Range of intracuff pressure (cm H_2_O)	23.1–34.0	21.8–31.3	23.1–32.6
Change from neutral position (cm H_2_O)	2.7 [1.4–4.1] ^c,d^	0 [0–2.7] ^b^	1.4 [0–2.7] ^b^
Number that decreased from neutral position	3 (6%)	10 (20%)	4 (8%)
Number with no change	7 (14%)	16 (32%)	15 (30%)
Number that increased from neutral position	40 (80%) ^c,d^	24 (48%) ^b^	31 (62%) ^b^
Number of cuffs with intracuff pressure <20 cm H_2_O	0 (0%)	0 (0%)	0 (0%)
Number of cuffs with intracuff pressure 20–30 cm H_2_O	44 (88%)	48 (96%)	47 (94%)
Number of cuffs with intracuff pressure >30 cm H_2_O	6 (12%)	2 (4%)	3 (6%)

Data are presented as median [IQR] or numbers (percentages). ^a^ *p* < 0.016 vs. neutral position. ^b^ *p* < 0.016 vs. flexed position. ^c^ *p* < 0.016 vs. extended position. ^d^ *p* < 0.016 vs. rotated position.

**Table 4:** Changes in peak inspiratory pressure with head positioning when the initial intracuff pressure was set as 24.48 cm H_2_O (=18 mmHg) using a pressure monitoring device.

Parameter	Neutral	Flexion	Extension	Rotation
Peak inspiratory pressure (cm H_2_O)	21.0 [19.0–22.0]	21.0 [19.0–22.0]	20.0 [19.0–22.0] ^a^	20.5 [19.0–22.0] ^a^
Change from neutral position (cm H_2_O)		0.0 [0.0–0.0] ^c^	0.0 [−1.0–0.0] ^b^	0.0 [−1.0–0.0]

Data are presented as median [IQR]. ^a^ *p* < 0.016 vs. neutral position. ^b^ *p* < 0.016 vs. flexed position. ^c^ *p* < 0.016 vs. extended position.

**Table 5:** Correlation coefficient of peak inspiratory pressure changes to intracuff pressure changes with head positioning.

Parameter	Correlation Coefficient (r)	*p*-Value
Change from neutral to extension	0.198	0.169
Change from neutral to flexion	−0.107	0.461
Change from neutral to rotation	0.125	0.387

## Data Availability

The datasets generated for this study are available on request to the corresponding author. The data are not publicly available due to privacy reasons.

## Supplementary Materials

The following are available online at https://www.mdpi.com/article/10.3390/jcm10173910/s1, Checklist S1: STROBE Statement—checklist of items that should be included in reports of observational studies.

## References

[1] Seegobin R.D., van Hasselt G.L. (1984). Endotracheal cuff pressure and tracheal mucosal blood flow: Endoscopic study of effects of four large volume cuffs. Br. Med. J. (Clin. Res. Ed.).

[2] Bouadma L., Wolff M., Lucet J.C. (2012). Ventilator-associated pneumonia and its prevention. Curr. Opin. Infect. Dis..

[3] Liu J., Zhang X., Gong W., Li S., Wang F., Fu S., Zhang M., Hang Y. (2010). Correlations between controlled endotracheal tube cuff pressure and postprocedural complications: A multicenter study. Anesth. Analg..

[4] Combes X., Schauvliege F., Peyrouset O., Motamed C., Kirov K., Dhonneur G., Duvaldestin P. (2001). Intracuff pressure and tracheal morbidity: Influence of filling with saline during nitrous oxide anesthesia. Anesthesiology.

[5] Coorey A., Brimacombe J., Keller C. (1998). Saline as an alternative to air for filling the laryngeal mask airway cuff. Br. J. Anaesth..

[6] Rosero E.B., Ozayar E., Eslava-Schmalbach J., Minhajuddin A., Joshi G.P. (2018). Effects of Increasing Airway Pressures on the Pressure of the Endotracheal Tube Cuff During Pelvic Laparoscopic Surgery. Anesth. Analg..

[7] Okgun Alcan A., van Yavuz Giersbergen M., Dincarslan G., Hepcivici Z., Kaya E., Uyar M. (2017). Effect of patient position on endotracheal cuff pressure in mechanically ventilated critically ill patients. Aust. Crit. Care.

[8] Kako H., Goykhman A., Ramesh A.S., Krishna S.G., Tobias J.D. (2015). Changes in intracuff pressure of a cuffed endotracheal tube during prolonged surgical procedures. Int. J. Pediatr. Otorhinolaryngol..

[9] Kim J.E., Nam Y.T., Chae Y.H. (1999). The Effect of the Body Position and CO2 Gas Insufflation on Airway Pressure and Compliance in Normal Subjects during Laparoscopy or Pelviscopy. Korean J. Anesthesiol..

[10] Wu C.Y., Yeh Y.C., Wang M.C., Lai C.H., Fan S.Z. (2014). Changes in endotracheal tube cuff pressure during laparoscopic surgery in head-up or head-down position. BMC Anesthesiol..

[11] Aeppli N., Lindauer B., Steurer M.P., Weiss M., Dullenkopf A. (2019). Endotracheal tube cuff pressure changes during manual cuff pressure control manoeuvres: An in-vitro assessment. Acta Anaesthesiol. Scand..

[12] Nseir S., Lorente L., Ferrer M., Rouzé A., Gonzalez O., Bassi G.L., Duhamel A., Torres A. (2015). Continuous control of tracheal cuff pressure for VAP prevention: A collaborative meta-analysis of individual participant data. Ann. Intensive Care.

[13] Kim S. (2018). Comparison of the cuff pressures of a TaperGuard endotracheal tube during ipsilateral and contralateral rotation of the head: A randomized prospective study. Medicine.

[14] Komasawa N., Mihara R., Imagawa K., Hattori K., Minami T. (2015). Comparison of Pressure Changes by Head and Neck Position between High-Volume Low-Pressure and Taper-Shaped Cuffs: A Randomized Controlled Trial. BioMed Res. Int..

[15] Kim H.C., Lee Y.H., Kim E., Oh E.A., Jeon Y.T., Park H.P. (2015). Comparison of the endotracheal tube cuff pressure between a tapered- versus a cylindrical-shaped cuff after changing from the supine to the lateral flank position. Can. J. Anaesth..

[16] Tailleur R., Bathory I., Dolci M., Frascarolo P., Kern C., Schoettker P. (2016). Endotracheal tube displacement during head and neck movements. Observational clinical trial. J. Clin. Anesth..

[17] Kako H., Krishna S.G., Ramesh A.S., Merz M.N., Elmaraghy C., Grischkan J., Jatana K.R., Ruda J., Tobias J.D. (2014). The relationship between head and neck position and endotracheal tube intracuff pressure in the pediatric population. Paediatr. Anaesth..

[18] Jordi Ritz E.M., Von Ungern-Sternberg B.S., Keller K., Frei F.J., Erb T.O. (2008). The impact of head position on the cuff and tube tip position of preformed oral tracheal tubes in young children. Anaesthesia.

[19] Hartrey R., Kestin I.G. (1995). Movement of oral and nasal tracheal tubes as a result of changes in head and neck position. Anaesthesia.

[20] Hall C.E., Shutt L.E. (2003). Nasotracheal intubation for head and neck surgery. Anaesthesia.

[21] Kako H., Alkhatib O., Krishna S.G., Khan S., Naguib A., Tobias J.D. (2015). Changes in intracuff pressure of a cuffed endotracheal tube during surgery for congenital heart disease using cardiopulmonary bypass. Paediatr. Anaesth..

[22] Brimacombe J., Keller C., Giampalmo M., Sparr H.J., Berry A. (1999). Direct measurement of mucosal pressures exerted by cuff and non-cuff portions of tracheal tubes with different cuff volumes and head and neck positions. Br. J. Anaesth..

[23] Yamanaka H., Tsukamoto M., Hitosugi T., Yokoyama T. (2018). Changes in nasotracheal tube depth in response to head and neck movement in children. Acta Anaesthesiol. Scand..

[24] Bernet V., Dullenkopf A., Cannizzaro V., Stutz K., Weiss M. (2006). An in vitro study of the compliance of paediatric tracheal tube cuffs and tracheal wall pressure. Anaesthesia.

[25] Matsuki Y., Matsuura N., Ichinohe T. (2016). Effect of Cuff Pressure Elevation on Internal Diameter of Tracheal Tube in Simulated Trachea. Bull. Tokyo Dent. Coll..

[26] Calder A., Hegarty M., Erb T.O., von Ungern-Sternberg B.S. (2012). Predictors of postoperative sore throat in intubated children. Paediatr. Anaesth..

[27] Özcan A.T.D., Döğer C., But A., Kutlu I., Aksoy Ş.M. (2018). Comparison of endotracheal tube cuff pressure values before and after training seminar. J. Clin. Monit. Comput..

[28] Giusti G.D., Rogari C., Gili A., Nisi F. (2017). Cuff pressure monitoring by manual palpation in intubated patients: How accurate is it? A manikin simulation study. Aust. Crit. Care.

[29] Michlig S.A. (2013). Anaesthetic staff cannot identify extremely high tracheal tube cuff pressures by palpation of the pilot balloon. Br. J. Anaesth..

[30] Pisano A., Verniero L., Galdieri N., Corcione A. (2019). Assessing the correct inflation of the endotracheal tube cuff: A larger pilot balloon increases the sensitivity of the ‘finger-pressure’ technique, but it remains poorly reliable in clinical practice. J. Clin. Monit. Comput..

[31] Pisano A. (2017). Pitfalls From Physics: Why We Can’t “Feel” the Tube Cuff Pressure With Our Fingers. Anesth. Analg..

[32] Spapen H.D., Suys E., Diltoer M., Stiers W., Desmet G., Honoré P.M. (2017). A newly developed tracheal tube offering ‘pressurised sealing’ outperforms currently available tubes in preventing cuff leakage: A benchtop study. Eur. J. Anaesthesiol..

